# Evaluation of Nanoparticles Covalently Bound with BODIPY for Their Photodynamic Therapy Applicability

**DOI:** 10.3390/ijms25063187

**Published:** 2024-03-10

**Authors:** Miryam Chiara Malacarne, Enrico Caruso, Marzia Bruna Gariboldi, Emanuela Marras, Gianluca Della Bitta, Orlando Santoro, Alan Simm, Rong Li, Calum T. J. Ferguson

**Affiliations:** 1 Department of Biotechnology and Life Sciences (DBSV), University of Insubria, Via J.H. Dunant 3, 21100 Varese, Italy; mc.malacarne1@uninsubria.it (M.C.M.); enrico.caruso@uninsubria.it (E.C.); marzia.gariboldi@uninsubria.it (M.B.G.); emanuela.marras@uninsubria.it (E.M.); gianluca.dellabitta@libero.it (G.D.B.); 2 Faculty of Sciences, Byrom Street Campus, Liverpool John Moores University, Liverpool L3 3AF, UK; a.m.simm@ljmu.ac.uk; 3 Max Planck Institute for Polymer Research, Ackermannweg 10, 55128 Mainz, Germany; lirong@mpip-mainz.mpg.de (R.L.); ferguson@mpip-mainz.mpg.de (C.T.J.F.)

**Keywords:** BODIPY, PDT, nanoparticles, singlet oxygen, death mechanism, cellular uptake

## Abstract

Photodynamic therapy (PDT) relies on the combined action of a photosensitizer (PS), light at an appropriate wavelength, and oxygen, to produce reactive oxygen species (ROS) that lead to cell death. However, this therapeutic modality presents some limitations, such as the poor water solubility of PSs and their limited selectivity. To overcome these problems, research has exploited nanoparticles (NPs). This project aimed to synthesize a PS, belonging to the BODIPY family, covalently link it to two NPs that differ in their lipophilic character, and then evaluate their photodynamic activity on SKOV3 and MCF7 tumor cell lines. Physicochemical analyses demonstrated that both NPs are suitable for PDT, as they are resistant to photobleaching and have good singlet oxygen (^1^O_2_) production. In vitro biological analyses showed that BODIPY has greater photodynamic activity in the free form than its NP-bounded counterpart, probably due to greater cellular uptake. To evaluate the main mechanisms involved in PDT-induced cell death, flow cytometric analyses were performed and showed that free BODIPY mainly induced necrosis, while once bound to NP, it seemed to prefer apoptosis. A scratch wound healing test indicated that all compounds partially inhibited cellular migration of SKOV3 cells.

## 1. Introduction

A highly selective and limited invasive treatment, which can be an alternative as well as an adjuvant to traditional oncological therapies, is PDT, which can be implemented both in the oncology field and other therapeutic fields (dermatological example) [1].

The mechanism of PDT involves the topical or systemic administration of a PS, which will accumulate in the tumor tissue, and will then be activated with a specific wavelength [2]. The activation of PS and its reaction with tissue oxygen will cause these three components, individually safe, to exhibit photocytotoxicity, generating ROS. This means that there is a selective destruction of only the tumor tissue.

Due to the insufficient light flux and the risk of damage to superficial tissues, its use against deep tumors is still limited today [3].

The photochemical reactions bring to triplet excited state formation with ROS production, which at the cellular level, can generate irreversible damage and cell death as the main consequence.

PDT-induced necrosis, apoptosis, and autophagy depend on PS’s properties and concentrations, light dose, and tissue oxygen. Tissue specificity is possible due to a preferential accumulation of PS in neoplastic tissues rather than in healthy ones.

Among the advantages of PDT, are the ability to not induce drug resistance, reduced invasiveness, the selective distribution of the PS, which allows for the treatment to be repeated without incurring side effects, and the possibility of directing the light thanks to the use of endoscopes.

Clinical research on PDT combined with chemotherapy, radiotherapy, and immunotherapy has shown increased therapeutic performance compared to single treatments [3].

To identify the ideal PS, various generations of PS have occurred over the years. The first-generation PSs included natural-origin molecules belonging to the porphyrin family (for example, Photofrin^®^ Concordia Laboratories Inc., Senningerberg, Luxembourg), which presented some disadvantages. The second generation of PSs was thus developed, made up of synthetic molecules belonging mainly to the porphyrin and phthalocyanine family and, more recently, to BODIPY.

BODIPYs, the acronym for 4,4-difluoro-4-bora-3a,4a-diaza-s-indacene, are molecules that possess interesting characteristics, including high molar extinction coefficient (ε) in the visible region, high fluorescence quantum yield (Φ), high stability to variations in pH, and the polarity of the solvent [4]. However, when BODIPYs are intended as PSs, the high quantum efficiency of fluorescence should be inhibited. To modify the absorption and emission characteristics, appropriate substituents can be introduced on the carbon atoms of the pyrrole ring. BODIPYs also present a high resistance to the well-known phenomenon of photobleaching, and are generally lipophilic molecules [5]; the degree of lipophilicity can, however, be modified by introducing substituents in the meso position of the molecules [6]. Currently, BODIPYs are mainly used as chemosensors, fluorescent switches, and biological probes [7,8,9].

The conjugation of PSs with inorganic and organic polymers, NPs, liposomes, peptides and carbohydrates [10,11,12,13] led to third-generation PSs [14], with improved selectivity, specificity, and better intracellular accumulation in tumor tissue [15].

One of the advantages of using NPs is related to the unique microenvironment characteristics of tumor tissue. Rapid tumor cell growth results in unusual cell sizes and creates an acidic environment different from that of normal tissues [16], characterized by the presence of abnormal vascularization with large fenestrations and poor lymphatic drainage [17]. These peculiarities are the basis of the so-called Enhanced Permeability and Retention effect (EPR), which favors the accumulation of macromolecules, of appropriate dimensions, in the vascularized tissue of the tumor [18,19].

In this manuscript, we reported the unprecedented covalent functionalization of NPs bearing hydrophilic and lipophilic polymerizable pendants with a suitably modified PS belonging to the BODIPY family. In particular, to obtain the NPs tested in this study, BODIPY-bearing syringaldehyde in the meso position was synthesized. The lipophilic component was benzyl alcohol derivative, while the hydrophilic one was made up of glycerol derivative. The in vitro phototoxic effect was evaluated on human breast cancer (MCF7) and ovarian cancer (SKOV3) cell lines. To investigate the photodynamic effects of these new NPs, cellular uptake, the induction of ROS production, and cell death mechanisms (apoptosis, necrosis and autophagy) were also evaluated, along with their ability to inhibit cell migration.

## 2. Results and Discussion

### 2.1. Chemical Analyses

**Figure 1:**
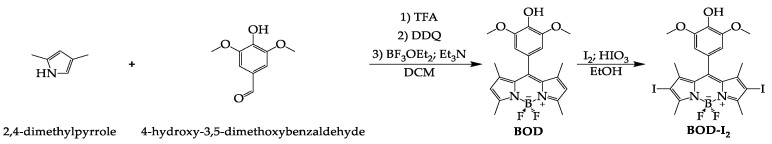
Synthesis scheme for the preparation of **BOD** and its iodinated derivative **BOD-I_2_**.

The procedure to obtain BODIPY has been reported by Akkaya e Liu [20,21]. The reaction involved the condensation of two units of 2,4-dimethylpyrrole with 4-hydroxy-3,5-dimethoxybenzaldehyde which provides the methenic carbon that acts as a bridge between the two rings to give dipyrromethane. This condensation was conducted in dichloromethane (DCM) under a N_2_ atmosphere using trifluoroacetic acid (TFA) as an acid catalyst. Subsequently, dipirromethane was oxidized by adding a slight excess of 2,3-Dichloro-5,6-dicyano-1,4-benzoquinone (DDQ), with the consequent formation of dipyrrolylmethene. BF_3_·Oet_2_ was added to the solution, in the presence of Et_3_N, which allows for the formation of the desired PS (**BOD**) (Figure 1). The intense fluorescent, yellow-colored **BOD** was recovered through a chromatographic column, with a yield of 13.5%.

A heavy atom introduction, such as iodine, in the β-position, is necessary to quench the fluorescence and improve the intersystem crossing (ISC) to achieve an increased production of ^1^O_2_ [22]. An iodization reaction was performed according to a modification developed in our laboratory of the Nagano method [6]. This reaction involved the use of I_2_ and HIO_3_ in EtOH at room temperature (RT) overnight (ON). The product (**BOD-I_2_**), after a first treatment with thiosulfate to remove the excess iodine, was purified by chromatographic column (yield 54.8%) (Figure 1).

**BOD-I_2_** and **BOD** monomers were synthesized following a procedure reported previously [23]; in a dried Schlenk tube **BOD-I_2_** or **BOD**, triethylamine and anhydrous DCM were stirred for 10 min and cooled by an ice bath. A slow acryloyl chloride addition was performed, and the reaction was stirred ON at RT. The solvent was removed, and pinky powders of **BOD-I_2_** and **BOD** monomers were obtained.

**Figure 2:**
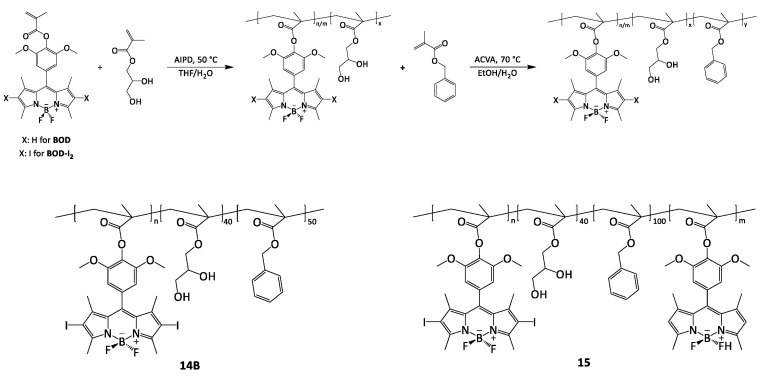
Synthesis scheme for the preparation of NPs and structure of **14B** and **15**.

PDT polymer NPs were obtained via the reversible addition–fragmentation chain transfer-mediated polymerization-induced self-assembly (RAFT-PISA) technique [24]. The hydrophilic macro-chain transfer agent (macroCTA) poly(2,3-dihydroxylpropylmethacrylate-co-**BOD-I_2_**) (P(PDHPMA-co-**BOD-I_2_**)) containing PS moieties has been prepared before blocking off a second hydrophobic benzyl methacrylate (BzMA) segment with/without **BOD**, which creates core-shell structured NPs (Figure 2).

These resulting PDT NPs are well-dispersed in aqueous conditions stabilized by the hydrophilic block (PDHPMA). Thw water compatibility and stability of the PS **BOD-I_2_** have been significantly enhanced by incorporating molecular **BOD-I_2_** into the PDHPMA block. Changes in pH within the range of 4–10 do not determine morphological or stability alterations of the NPs tested, as previously reported [25].

**Figure 3:**
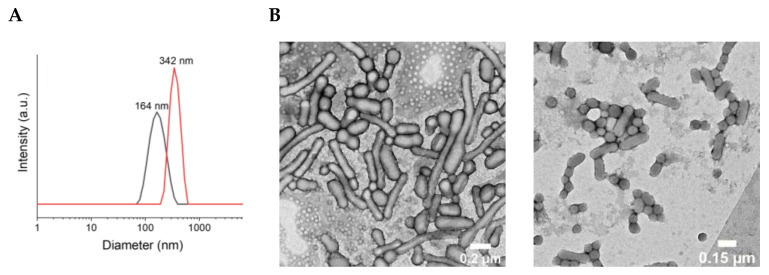
(**A**) DLS measurement of **14B** (red) and **15** (black) NPs dispersions in DIW with hydrodynamic diameters peaking at 164 nm (PDI = 0.236) and 342 nm (PDI = 0.086), respectively. (**B**) TEM images of **14B** (**left**) and **15** (**right**) NPs dispersion show a mixed morphology of spherical NPs and rods.

The hydrodynamic diameter of PDT N”s (c’ntered at 164 nm for **15** and 342 nm for **14B**) has been characterized by Dynamic Light Scattering (DLS) (Figure 3A). A mixed morphology of spherical NPs and nanorods was observed with transmission electron microscopy (TEM) (Figure 3B).

The molecular weight of PDT-NPs was examined through GPC (**14B**, Mn = 34.6 kDa, red) and **15** (Mn = 42.3 kDa) (Figure S1). Chemical compositions of these PDT NPs have been confirmed by ^1^H NMR (Figures S2 and S3), and Fourier-transform infrared spectroscopy (FTIR) (Figure S4); however, specific peaks for PS moieties were not observed due to the small loading quantity of sensitizer monomers. The FTIR spectrum exhibited an aliphatic backbone (−CH_2_− and −CH_3_) stretch vibration from 2775 to 3130 cm^−1^. Furthermore, typical signals at 1727, 1262, and 1174 cm^−1^ are ascribed to carbonyl C=O stretching, Ar-O stretching, and C−O stretching, respectively [26].

**Figure 4:**
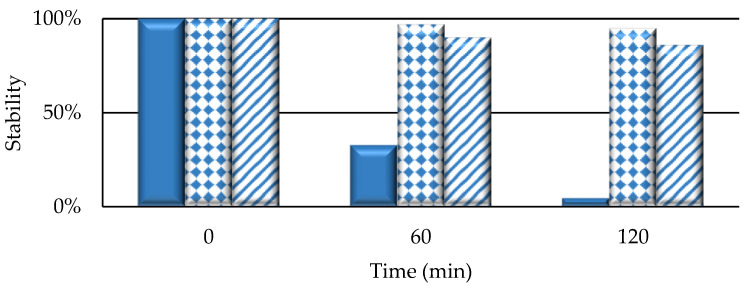
Residual stability of 10 µM solution of PSs in PBS 1X after 500 W tungsten halogen lamp irradiation for 2 h and subjected to spectrophotometric analysis (**BOD-I_2_**: full bar; **14B**: dotted bar; **15** (**BOD-I_2_**): striped bar).

In the presence of oxidizing species, primarily ^1^O_2_, PSs partially degraded [27]. Nevertheless, recent evidence suggests that this photodegradation is mainly induced by radicals (Type I reaction) [28]. The stability of iodinated BODIPY, both in free form and nanoformulated, was evaluated by irradiating a 1 × 10^−5^ M concentration in 1X phosphate-buffered saline (PBS) with a white tungsten halogen lamp for 2 h and then measuring the absorbance after 60 and 120 min. At the same time, the possible appearance of modified absorbance profiles was also monitored, the appearance of which could be indicative of the formation of different species. The results obtained through the photobleaching test are shown In Figure 4.

BODIPY in free form (**BOD-I_2_**) loses about 2/3 of its stability in the first hour of illumination and degrades almost completely at the end of the second hour (4% stability). The situation changed considerably when NPs containing the same molecule were considered; in fact, they showed high stability to photodegradation (stability > 85% after 2 h). Therefore, NPs make PS more stable, giving rise to a protective effect of the PS itself. The same protective effect was also observed by comparing the stability of the fluorescent compound **BOD** both in free form and bound to the NP (Figure S5). In addition, the results obtained did not evidence absorbance changes for the compounds analyzed, indicating the absence of the formation of other species during the photobleaching process.

To evaluate the ^1^O_2_ production rates, the indirect method based on 1,3-diphenylisobenzofuran (DPBF) degradation was used. This method involved the generation of ^1^O_2_ from each PS by 10 min irradiation with green LED and the evaluation of DPBF degradation by the produced ^1^O_2_. Degradation kinetics were then normalized with respect to Rose Bengal, a well-known producer of ^1^O_2_ [29].

Our results show that free **BOD-I_2_** has a relative rate of ^1^O_2_ production equal to 0.35, while an increase in this value was observed when **BOD-I_2_** was linked to NPs (0.70 for **14B** and 0.59 for **15**, respectively). The explanation could lie both in the BODIPY structure itself and the hydroxyl group in the phenyl ring para position. In NP derivatives, this group is used to form ester bonds with carboxyl residues of the NPs [30], while in **BOD-I_2_**, it is free and could exert an antioxidant effect, thus leading to lower relative rates of ^1^O_2_, compared to the NP derivatives [31].

Data indicate that NPs do not have any shielding effect on BODIPY in ^1^O_2_ production; in addition, ^1^O_2_ does not directly degrade the NPs themselves.

### 2.2. Biological Analyses

Biological studies were performed on the human ovarian carcinoma SKOV3 and breast adenocarcinoma MCF7 cell lines. To verify the selectivity of the studied compounds towards tumor cells, compared to non-tumorigenic cells, their photodynamic activity was also evaluated on the fibroblast cell line MRC-5. The MTT assay ([3-(4,5-dimethylthiazol-2-yl)-2,5-diphenyltetrazolium bromide]) was used to evaluate the effects on cell viability. This test involved treatment for 24 h with the three compounds, irradiation for 2 h (fluence: 158 J/cm^2^), and 24 h of incubation in drug-free medium. The IC_50_ values were obtained from the dose–response curves.

For all compounds, including unloaded NP, intrinsic cytotoxicity, evaluated by omitting the irradiation step and using concentrations of the compounds ten times higher than those used for tests in which irradiation was performed, was negligible in all cases (Figure S6).

**Figure 5:**
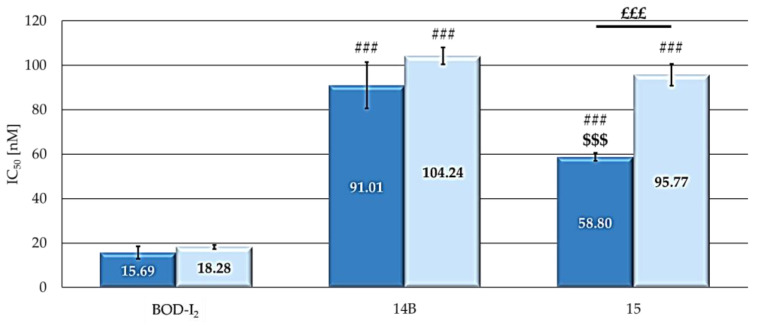
IC**_50_** values (**MCF7**: dark blue; **SKOV3**: pale blue) obtained following 24 h treatment with increasing concentrations of the studied compounds, 2 h irradiation under visible light of a 500 W halogen lamp, 24 h incubation in drug-free medium and MTT assay. Mean ± SD of 5 independent experiments. Statistics were performed using two-way ANOVA followed by Duncan’s post hoc test. ### *p* < 0.001 vs. **BOD-I_2_**; $$$ *p* < 0.001 vs. **14B**; £££ *p* < 0.001.

Figure 5 and Figure S7 show that **BOD-I_2_** has a greater photodynamic activity compared to the equivalent NP-bound derivatives, with the IC_50_ value being 4–6 times lower in the former. A possible explanation for this result could be related to what has been reported by other authors, who have observed that part of the oxidative action of the ^1^O_2_ in NPs could be used to degrade the polymers themselves [32], thus influencing the potency of NP formulations; however, the lowest ^1^O_2_ levels produced by the free BODIPY in the present study appear to disprove this hypothesis. Specifically analyzing the activity of the NP formulations, **14B** exerted similar photodynamic effects in both cell lines, while **15** was significantly more active in MCF7 cells. The lower potency of the NP-bounded derivatives should not be considered limiting; in fact, the excellent water solubility and the high photobleaching resistance might make these nanosystems suitable for in vivo applications where the EPR effect also becomes extremely important [33]. The possibility of preferential accumulation in tumor tissues associated with the possibility of decorating the NPs to implement specific targeting systems should favor the use of these NPs for clinical applications compared to free BODIPY [34].

Interestingly, no photodynamic activity was observed on the fibroblast cell line MRC-5 treated in the same conditions used for the tumor cell lines (Figure S8), indicating a selectivity of the tested compounds for tumor cells.

**Figure 6:**
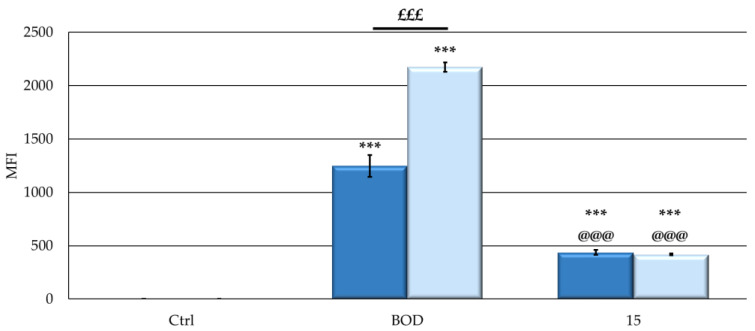
Cellular uptake expressed in mean fluorescence intensity (MFI) values (**MCF7**: dark blue; **SKOV3**: pale blue) obtained following 24 h treatment with 100 nM of **BOD** or **15**. After exposure, cells were trypsinized, resuspended in PBS, and analyzed with FACSCalibur. Absorption was quantified in arbitrary units (a.u.) based on MFI, by collecting PS fluorescence through a 530 nm band-pass filter. PS treatment was omitted in the control samples. Mean ± SD of 5 independent experiments. Statistics were performed using two-way ANOVA followed by Duncan’s post hoc test. *** *p* < 0.001 vs. **Ctrl**; @@@ *p* < 0.001 vs. **BOD**; £££ *p* < 0.001.

An extremely important characteristic related to good photodynamic effects is the ability of the PS to be internalized in cells, allowing for its subsequent oxidative effect [35,36]. Treatment of SKOV3 and MCF7cells for 24 h with an equimolar concentration (100 nM) of the fluorescent BODIPY analog (**BOD**) and **15**, in which the NPs were loaded with both fluorescent and iodinated BODIPYs, resulted in a significant increase in cellular uptake of the two compounds. In particular, **BOD** entered both cell lines at a higher extent, compared to **15** (Figure 6). The lower uptake of **15** could be explained considering that, due to the absence of specific target groups, only passive absorption is observed; therefore, NP dimensions, which are higher than free BODIPY ones, have a negative impact on their uptake.

In addition, **BOD** is absorbed differently by the two cell lines, while **15** showed similar uptake levels in both cell lines.

Thus, as reported by other authors for both porphyrins and boron-dipyrromethenic systems, our results indicate that the significantly higher cellular uptake of free BODIPY compared to those that are NP-bound could be the responsible for the different photodynamic activity observed [37,38]. However, MCF7 cells appear to be particularly sensitive to PDT with both formulations, compared to the SKOV3 cell line. Indeed, despite being less absorbed by MCF7 cells, free BODIPY had the same photodynamic activity in both MCF7 and SKOV3 cells. Furthermore, greater photodynamic activity was observed in the MCF7 following treatment with **15** even if a comparable uptake was detected in the two cell lines. Similar behavior for MCF7 cells has been observed by other authors, including Lamch [39] and Li [40]. Nevertheless, others evidenced opposite results [41], suggesting that the different stress responses induced by the photodynamic process could be strictly linked to the type of both the PS investigated and the cell lines [42,43].

In PDT, ^1^O_2_ and ROS trigger tumor cell death, and light is used to stimulate the PS to induce their production [44,45]. As reported above, the free and bound **BOD-I_2_** in the two NP derivatives showed a good degree of ^1^O_2_ production. To detect intracellular production of ROS, cells were treated with equitoxic concentrations of PSs for 24 h and irradiated for 2 h prior to incubation with dichloro-dihydro-fluorescein diacetate (DCHF-DA). The presence of ROS, produced during irradiation, allows for the oxidation of dichloro-dihydro-fluorescein (DCHF), obtained by the internalized DCHF-DA cleavage, to dichlorofluorescein (DCF). The latter can be detected using a fluorescence microscope (Figure S9) [46,47,48,49]. The ROS production rates were measured with ImageJ 1.49V software (National Institute of Health).

**Figure 7:**
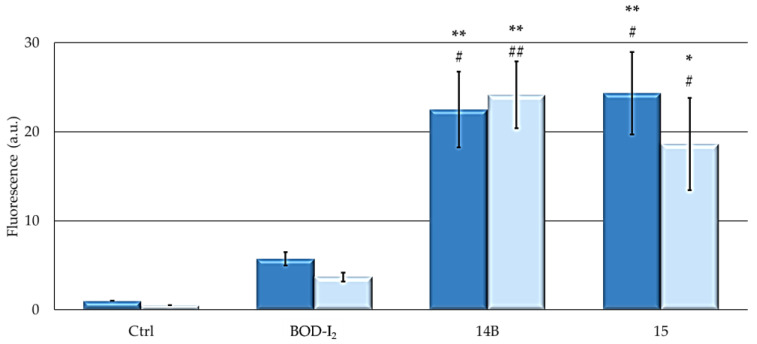
ROS expressed in a.u. of fluorescence (**MCF7**: dark blue; **SKOV3**: pale blue) obtained following 24 h treatment at each PS IC_50_, 2 h of irradiation in PS-free PBS, and 30 min incubation with 10 µM of DCFH-DA. Fluorescence microscopy was used to detect the production of ROS (Ex: 488 nm; Em: 520 nm) and the ROS production rate was measured with ImageJ software. For control samples, treatment with PS was omitted. Mean ± SD of 5 independent experiments. Statistics were performed using two-way ANOVA followed by Duncan’s post hoc test. ** *p* < 0.01; * *p* < 0.05 vs. **Ctrl**, ## *p* < 0.01; # *p* < 0.05 vs. **BOD-I_2_.**

Figure 7 shows that all formulations can induce a significant increase in ROS levels compared to control and a similar extent of increased ROS levels in both cell lines. Furthermore, the two NP formulations induced higher ROS production than the free BODIPY.

ROS and ^1^O_2_ are the main causes of cell death following PDT. Several authors have linked ROS production to extrinsic (death receptor-dependent) [50,51,52] and intrinsic (mitochondrial-dependent) [53,54,55] apoptosis. However, high levels of ROS have also been correlated to necrosis [56,57,58]. Finally, ROS are also responsible for autophagy [59,60,61]. Thus, in the presence of high ROS levels, it is not possible to precisely know which type of cell death mechanism is the main protagonist. It therefore becomes very important to specifically evaluate the various types of cell death induced by PDT with new PSs.

**Figure 8:**
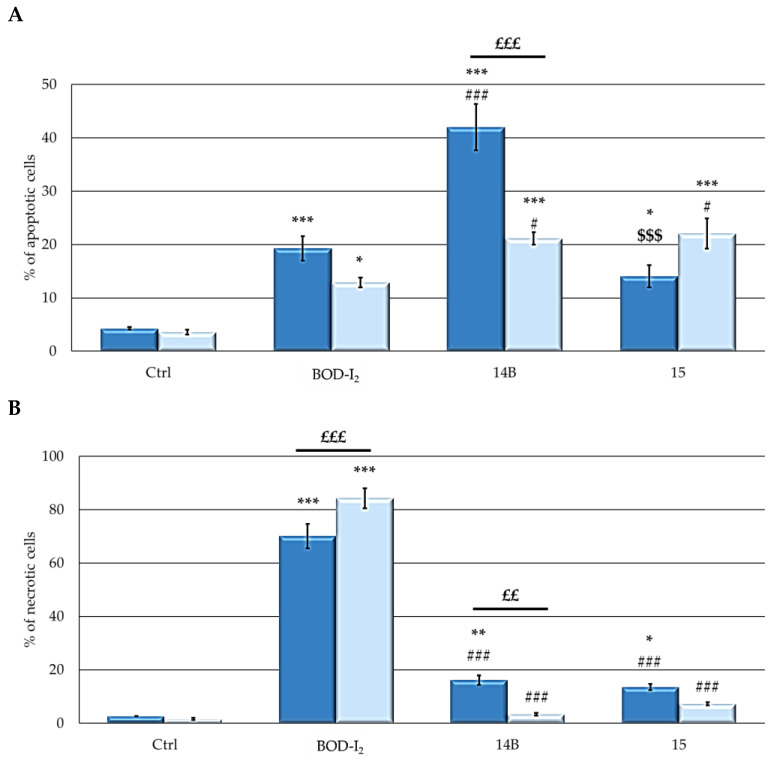
Percentage of apoptotic (**A**) and necrotic (**B**) cells (**MCF7**: dark blue; **SKOV3**: pale blue). Cells were treated for 24 h with PS at the respective IC_50_, and irradiated for 2 h in PBS. After 24 h, the percentage of apoptotic or necrotic cells was detected with FACSCalibur. To evaluate apoptosis, cells were fixed and resuspended in a PBS solution containing PI (50 µg/mL) and Rnase A (30 U/mL). For the evaluation of necrosis, the EtOH fixation was omitted. For both analyses in the control samples, treatment with PS was omitted. The fluorescent emission of PI was collected through a 575 nm band-pass filter. Mean ± SD of 5 independent experiments. Statistics were performed using two-way ANOVA followed by Duncan’s post hoc test *** *p* < 0.001; ** *p* < 0.01; * *p* < 0.05 vs. **Ctrl**; ### *p* < 0.001; # *p* < 0.05 vs. **BOD-I_2_**; $$$ *p* < 0.001 vs. **14B**; £££ *p* < 0.001; ££ *p* < 0.01.

Figure 8A shows a significant increment in the percentages of apoptotic cells induced by all compounds. However, the behavior of the studied formulations was different in the two cell lines. In MCF7 cells, **14B** induced the highest level of apoptosis (40%), while **BOD-I_2_** and **15** showed significantly lower percentages of apoptosis (15–20%). In SKOV3 cells, both NP derivatives induced higher percentages of apoptosis (20–25%), compared to **BOD-I_2_** (15%). On the other hand, all compounds induced an increase in the percentages of necrotic cells, compared to control. Among the PSs, in both cell lines, **BOD-I_2_** induced the highest level of necrosis (70–85%) while the treatment with both NPs resulted in significantly lower percentages of necrosis (<15%).

Overall, these data indicate that free BODIPY preferentially induced necrosis as the main cell death mechanism following PDT, while NPs induced mainly apoptosis.

In the absence of irradiation, none of the compounds triggered apoptotic or necrotic responses.

Autophagic cell death has been also indicated as a PDT-induced cell death mechanism [62,63,64]. To assess the degree of autophagy induced by compounds, Western blot analysis was performed to detect the presence of LC3-II, a known marker for autophagy.

**Figure 9:**
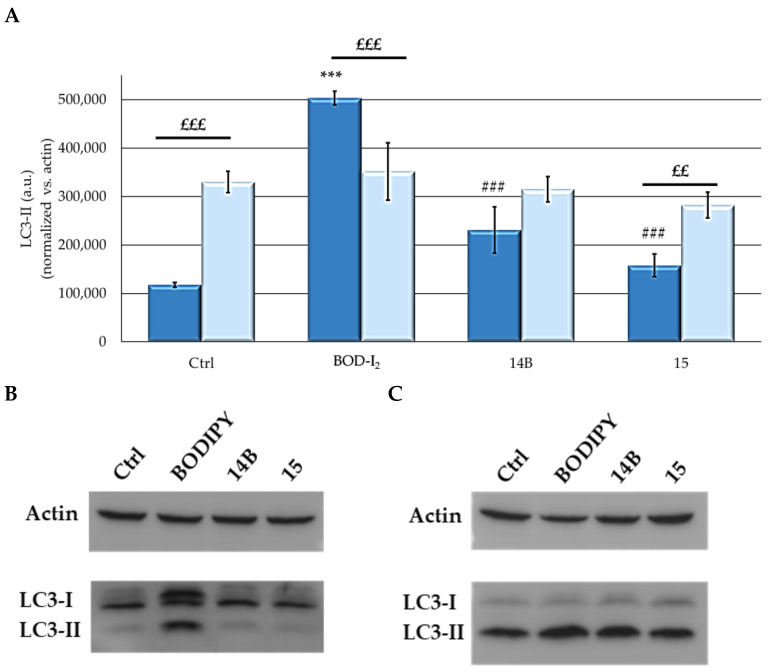
Densitometric analyses of LC3-II levels in two cell lines tested (**A**) and representative immunoblot images in **MCF7** (**B**) and **SKOV3** (**C**) cells. Cells were treated for 24 h with PS at the respective IC_50_ (PS treatment omitted in control) and irradiated for 2 h in PBS. After 24 h of incubation in drug-free medium, cells were lysed, and the protein concentration of each lysate was detected via the BCA test. SDS-page and Western blot analysis were performed, and LC3-II levels were evaluated. Histograms relative to densitometric analysis, performed with ImageJ, of all the experiments and Western blot representative images are reported. Mean ± SD of 2 independent experiments. Statistics were performed using two-way ANOVA followed by Bonferroni’s post hoc test. *** *p* < 0.001 vs. **Ctrl**; ### *p* < 0.001 vs. **BOD-I_2_**; £££ *p* < 0.001; ££ *p* < 0.01.

The two lines responded differently to treatment with BOD-I2, as indicated by the histograms and the representative Western blot images reported in Figure 9. While BOD-I2 induced increased levels of LC3-II in MCF7 cells compared to the control, the level of this protein did not change in the SKOV3 cell line. Concerning the other two compounds, both cell lines responded similarly to the treatments.

**Figure 10:**
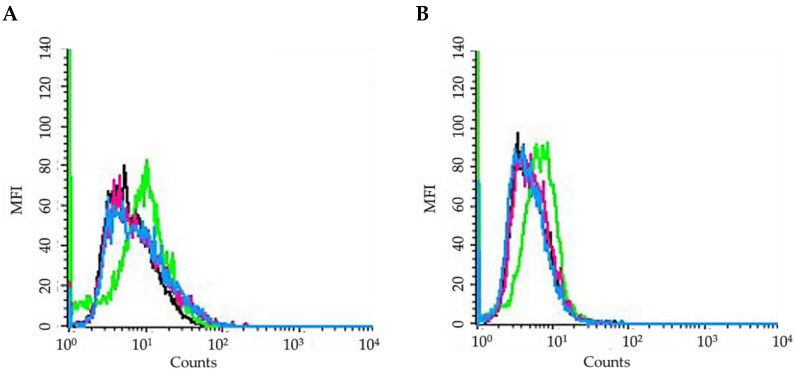
AO fluorescence expressed as MFI in MCF7 (**A**) and SKOV3 (**B**) cells (**Ctrl**: black line; **BOD-I_2_**: green line; **14B**: purple line; **15**: light blue line) treated for 24 h with equitoxic concentrations of PSs corresponding to the respective IC_50_ values, 2 h irradiation, 24 h incubation in drug-free medium and 15′ incubation at 37 °C in the presence of AO (1 µg/mL).

To confirm autophagy presence, flow cytometric evaluation of the acridine orange (AO) specific staining in MCF7 and SKOV3 cells following treatment with the PSs was performed. Figure 10 shows that, similarly to what was observed in Western blot analysis, only following **BOD-I_2_** treatment, a significant increase in AO fluorescence was observed in both cell lines, indicating the induction of autophagic cell death.

It is well known that along with cellular uptake, subcellular localization of a PS can account for its phototoxic effects [65].

**Figure 11:**
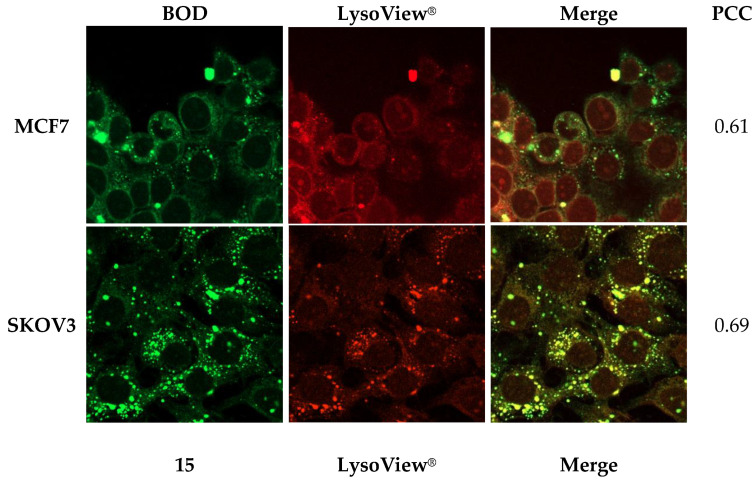

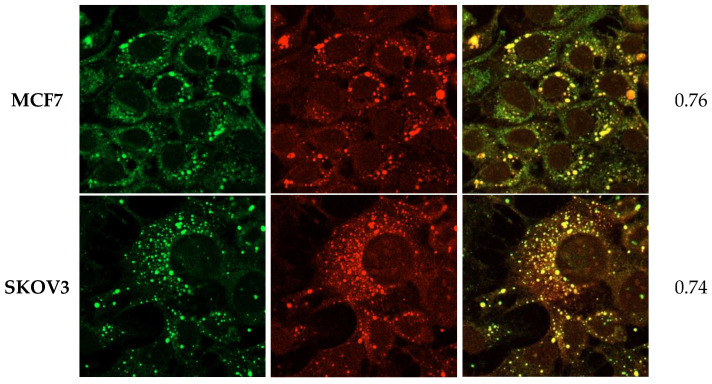
Intracellular **BOD** or **15** colocalization and PCC values. Cells were grown on coverslips for 48 h, treated with 25 μM of each PS (green fluorescence) and 2X of LysoView^®^ (red fluorescence). After 24 h, cells were fixed in 3% paraformaldehyde in H_2_O (pH = 7.4). Images were acquired using ZEISS LSM 900 with Airyscan 2 (Magnification 63×).

Figure 11 shows images related to the lysosomal colocalization of unbound **BOD** and **15** after 24 h exposure with the LysoView^®^ (Biotium Inc., Freemont, CA, USA). Pearson’s correlation coefficient (PCC) is used to measure the correlation between two sets of data [66]. In this experiment, PCC was used to measure the colocalization of our PSs, and the results were the following: **BOD** in MCF7 0.61, in SKOV3 0.69; **15** in MCF7 0.76, in SKOV3 0.74. Lysosomal localization is higher when BODIPY is bound to the NP. Furthermore, a diffuse plasmatic signal that cannot be attributed to distinct organelles was observed.

The high **BOD** localization at lysosomal level in the two tumor cell lines could explain the preferential necrotic cell death mechanism due to PDT treatment [67]. However, it is more difficult to explain the correlation between a high lysosomal localization of **15** and its limited percentage of necrosis at IC_50_ concentration.

A possible problem with the experimental setting we have used for this last evaluation is related to the fact that to obtain a good resolution of the fluorescence images, it was necessary to treat the cells with high concentrations of the PSs. At these concentrations, PSs could accumulate in both primary and low affinity sites [68]; consequently, it may be difficult to identify the priority site of cellular localization.

Metastasis refers to the process by which one or more cancer cells detach from the primary tumor, enter the bloodstream, and invade new organs (micro-metastases) where they will proliferate uncontrollably into macro-metastases [69,70]. Among the two cell lines, only the ovarian cancer cell SKOV3 possesses intrinsic migratory ability associated with the capability to cause metastasis [69,71,72,73]. It is reported that a fair number of PSs can block the migratory activity of cancer cells [74,75,76]. Cell migration is related to changes in the cytoskeleton and its components. PDT-linked oxidative stress causes changes in cytoskeletal components and, therefore, may affect the ability of cells to migrate and their migration rate [77].

The ability of the studied PSs to inhibit cell migration and the migration rate in response to PDT was evaluated by the scratch wound healing assay, in which a scratch was performed on a confluent monolayer, and the percentage of scratch closure as well as the migration rate were evaluated [78].

**Figure 12:**
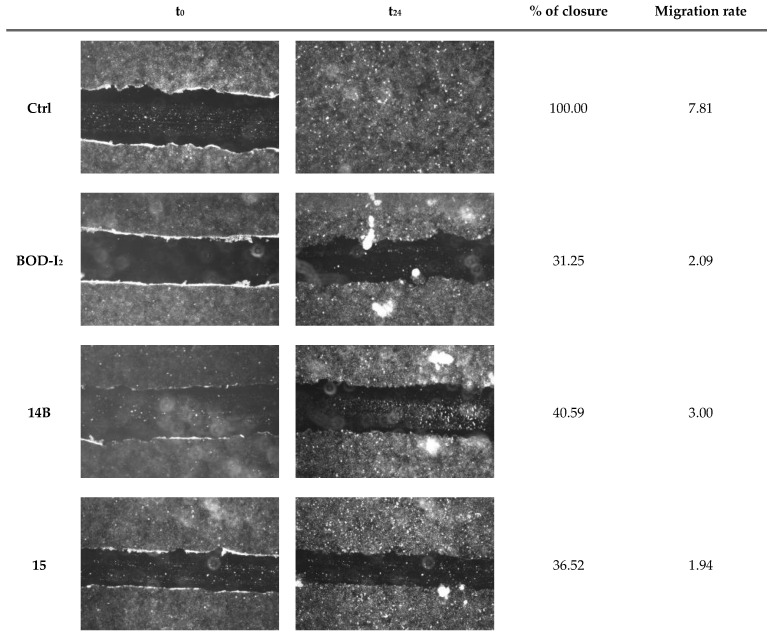
Migratory activity of SKOV3 cells following treatment with **BOD-I_2_**, **14B**, **15** and **PDT.** Cells were grown for 48 h before they were treated with the PSs at IC_25_ concentrations, scratched with a pipette tip, irradiated for 2 h, and placed in drug-free medium for 24 h. Pictures of the scratch wound were taken immediately following the wound making and after 24 h, through a camera connected to an Olympus IX81 microscope (Magnification 4×).

When SKOV3 cells were treated but not subjected to the irradiation step (not subjected to PDT), no effects on their migratory ability or their migration rate, compared to control cells, were observed (Figure S10). Instead, following treatment and PDT, all the compounds inhibited cell migration and caused a half-reduction of the migration rate (Figure 12).

## 3. Materials and Methods

### 3.1. Chemistry

#### 3.1.1. Chemicals and Experimental Instruments

^1^H NMR, UV-Vis absorption, elemental analysis, HPLC and MS analysis were performed as previously reported [79].

4-Hydroxy-3,5-dimethoxybenzaldehyde (M.W.: 182.17 g/mol), 2,4-dimethylpyrrole (M.W.: 95.14 g/mol, d: 0.92 g/mL), 4,4′-azobis(4-cyanovaleric acid) (ACVA, M.W.: 280.28 g/mol), 2,2′-azobis[2-(2-imidazolin-2-yl)propane]dihydrochloride (AIPD, M.W.: 323.27 g/mol), BzMA (M.W.: 176.21 g/mol, d: 1.04 g/mL), 4-((((2-carboxyethyl)thio)carbonothioyl)thio)-4-cyanopentanoic acid (CCCP, M.W.: 307.41 g/mol), glycidyl methacrylate (GMA, M.W.: 142.15 g/mol, d: 1.04 g/mL), and all solvents used in the synthesis, as well as those used for the analyzes, were commercial products used as received by Merck KgaA (Darmstadt, Germany, EU). DCM, used in the synthesis of BODIPYs, was freshly distilled directly into the reaction flask. The petroleum ether (ETP) employed as eluent for column chromatography consisted of a 40–60 fraction.

For all BODIPYs, 1 mM stock solutions in dimethyl sulfoxide (DMSO) were prepared. Water-soluble NPs **14B** and **15** had an iodinated compound concentration of 0.52 mM and 0.50 mM, respectively.

For the chemical analyses that required the use of light sources, a green LED light source or a 500 W tungsten white halogen lamp was used. The green LED light source consists of 12 LEDs, each of 1 W, for a total of 12 W. The control unit was connected to the lamp via an RJ45 connection and was powered by a constant current of 350 mA. The source had an irradiance equal to 3.036 mW/cm^2^, equal to 1.82 J/cm^2^ of fluence for 10 min of illumination.

The irradiation device with a tungsten lamp was placed above the area to be irradiated at a distance such as to produce a homogeneous irradiation area; the lamp had an irradiance of 22 mW/cm^2^, equal to 158 J/cm^2^ of fluence for 2 h of irradiation [79].

#### 3.1.2. Synthesis of BODIPYs

*4,4′-difluoro-1,3,5,7-tetramethyl-8-(4-hydroxy-3,5-dimethoxyphenyl)-4-bora-3a,4a-diaza-s-indacene* (**BOD**)

Then, 3.82 mmol of 4-hydroxy-3,5-dimethoxybenzaldehyde (696 mg) and 8.14 mmol of 2,4-dimethylpyrrole (840 μL) were reacted as previously described [11]. The raw material was purified by column chromatography (SiO_2_, DCM: ETP, 7:3), affording 206 mg (0.515 mmol, yield: 13.5%) of the desired compound (**BOD**) as orange needles. Chemical formula: C_21_H_23_BF_2_N_2_O_3_, M_w_ = 400.23 g/mol. UV–Vis: (DCM) 502 nm (ε= 163,000); (H_2_O) 498 nm (ε = 69,900). Φ_fl_: (DCM) 517 nm= 0.042. ^1^H NMR (CDCl_3_) δ: 1.55 (s, 6H); 2.58 (s, 6H); 3.89 (s, 6H); 6.02 (s, 2 h); 6.55 (s, 2 h). MS (ESI): M+ found: 401.18. HPLC retention time: 6′25″ (98%). Anal. Calc.: C, 63.02%; H, 5.79%; N, 7.00% Found: C, 63.58%; H, 5.85%; N, 7.07.

*4,4′-difluoro-2,6-diiodo-1,3,5,7-tetramethyl-8-(4-hydroxy-3,5-dimethoxyphenyl)-4-bora-3a,4a-diaza-s-indacene* (**BOD-I_2_**)

Then, 0.475 mmol of **BOD** (190 mg) was dissolved in 50 mL of EtOH in the presence of 2.38 mmol di I_2_ (602 mg; M.W.: 254 g/mol) e 2.38 di HIO_3_ (418 mg; M.W.: 176 g/mol) and reacted as previously reported [11]. The crude product was purified by column chromatography (SiO_2_, DCM: ETP, 7:3), affording 106 mg of **BOD-I_2_** (0.37 mmol; yield: 54.80%) as pink needles. Chemical formula: C_21_H_21_BF_2_I_2_N_2_O_3_, M_w_ = 652.03 g/mol. UV–Vis: (DCM) 534 nm (ε = 49,900); (H_2_O) 542 nm (ε = 23,600). ^1^H NMR (CDCl_3_) δ: 1.62 (s, 6H); 2.65 (s, 6H); 3.91 (s, 6H); 6.51 (s, 2 h). MS (ESI): M+ found: 652.97. HPLC retention time: 7′38″ (97%). Anal. Calc.: C, 63.68%; H, 3.25%; N, 4.30% Found: C, 64.02%; H, 3.28%; N, 4.33.

#### 3.1.3. Oxidation of GMA

GMA monomers (4.0 g) in 16.0 g DI H_2_O (20 wt% of GMA) were charged into a 40 mL glass vial under oxygen atmosphere. The reaction solution was heated up to 70 °C for 24 h. The oxidized GMA monomers were used for macro-CTA preparation without further purification.

#### 3.1.4. Preparation of PGMA-PS Macro-CTA

CCCP RAFT agent (13.8 mg, 0.045 mmol), GMA (719.9 mg, 45 mmol) in DI H_2_O (2.88 g), AIPD (2.9 mg, 0.009 mmol; CCCP/AIPD molar ratio = 5.0), and PS (0.045 mmol) were charged into a 40 mL glass vial and mixed with tetrahydrofuran (THF) (3.6 g) to obtain 10 wt% solid content in THF/H_2_O mixture. The mixture was degassed with Ar before heated up to 50 °C for 1.5 h. The crude macro-CTA was dialyzed against THF/H_2_O for 3 days.

#### 3.1.5. NP Synthesis

Synthesis of *LIR-10-14B* (**14B**)

PGMA- **BOD-I_2_** (138.9 mg, 0.019 mmol), BzMA (16.9 mg, 0.96 mmol), ACVA (1.1 mg, 0.0039 mmol) and ethanol/ H_2_O (50/50, 3 mL, 10 wt% solid content) were charged in a 20 mL glass vial and degassed with Ar for 10 min, before being placed into a heating block at 70 °C for 3 h. The obtained copolymer was analyzed by DMF GPC (Mn = 34.6 kDa, PMMA standards). ^1^H NMR analyzed in DMSO-d_6_ indicated less than 5% residual BzMA monomer. The resulting particles were purified via dialysis against DIW/Acetone for 3 days and DIW for 2 days. UV-Vis: (H_2_O) 542 nm (ε = 60,000).

Synthesis of *LIR-10-15* (**15**)

PGMA-**BOD-I_2_** (142 mg, 0.019 mmol), BzMA (173 mg, 0.98 mmol), PGMA-**BOD** (9 mg, 0.02 mmol), ACVA (1.10 mg, 0.0039 mmol) and ethanol/ H_2_O (50/50, 2.8 mL, 10 wt% solid content) were charged in a 20 mL glass vial and degassed with Ar for 10 min, before being placed into a heating block at 70 °C for 3 h. The resulting copolymer was analyzed by DMF GPC (Mn = 42.3 kDa, PMMA standards). ^1^H NMR analyzed in DMSO-d_6_ indicated less than 5% residual BzMA monomer. The resulting particles were purified via dialysis against DIW/Acetone for 3 days and DIW for 2 days. UV-Vis: (H_2_O) 507 nm (ε = 134,000), 541 nm (ε = 30,000). Φ_fl_: (H_2_O) 524 nm = 0.092.

#### 3.1.6. Photobleaching and ^1^O_2_ Generation

A 10 μM solution in 1X PBS was prepared for each sample. The solutions thus obtained were illuminated with a 500W tungsten halogen lamp for 2 h. At set times, samples were taken and subjected to spectrophotometric analysis. The percentage of photodegradation was calculated by the following Equation (1):

(1) $$\mathrm{Residual}\;\mathrm{stability}\;\left(\%\right)=\left(1-\frac{{\mathrm{Abs}}_{t}}{{\mathrm{Abs}}_{0}}\right)\times 100$$

The amount of ^1^O_2_ produced by the compounds was detected as previously reported [6].

### 3.2. Biology: Cell Cultures and Protocols

The breast cancer MCF7 and the ovarian cancer SKOV3 cell lines were maintained as reported [79]. For all experiments, unless indicated, 2 h irradiation with a 500 W white tungsten halogen lamp was used.

The effects of the PSs on cell viability were evaluated by the MTT assay as previously reported [79]. Cells were treated with different concentrations of **BOD-I_2_** (1 to 100 nM), **14B** (1 to 2000 nM), and **15** (1 to 1000 nM). The effects of PSs were also evaluated on a fibroblast cell line (MRC-5 grown in DMEM-F12) using concentrations of PSs from 1 to 1000 nM. The Infinite^®^ 200 PRO (Tecan, Bio-Rad Inc., Hercules, CA, USA) with a 590 nm filter was used to read the cell survival data. The data obtained were analyzed by non-linear regression using GraphPad PRISM 9.2.0 software (GraphPad Software Inc., San Diego, CA, USA); IC_50_ values were obtained.

To evaluate PS uptake, cells were exposed for 24 h to 100 nM of **BOD** or **15** and then worked up as reported [79]. All samples were analyzed with FACSCalibur, and data were analyzed with CellQuest PRO software(V6.0, BD Bioscences). Absorption was quantified in MFI, by collecting PS fluorescence through a 530 nm band-pass filter.

The fluorogenic probe DCHF-DA (InVitrogen Molecular Probes, ThermoFischer Scientific, Waltham, MA, USA) was used to evaluate intracellular generation of ROS using a reported method [80]. Olympus IX81 fluorescence microscopy, camera connected, was used to detect the production of ROS (Ex: 488 nm; Em: 520 nm). The ROS production rate, expressed as a.u. of fluorescence, was measured with ImageJ software [81].

The ability of the compounds to induce death through apoptosis or necrosis was evaluated with a flow cytometric analysis [79]. The induction of autophagy was evaluated by Western blot analysis to detect the autophagosomal marker LC3-II following the standard procedure used in our laboratory [79] and by evaluating acridine orange fluorescence through flow cytometric analysis, following PDT and incubation with 1 μg/mL AO.

Lysosomal localization was evaluated by fluorescence microscopy following coincubation with 25 μM of each PS and 2× of LysoView^®^ as reported [82]. Images were acquired using a ZEISS LSM 900 with an Airyscan 2 confocal microscope. Colocalization was quantified with PCC using the JaCOP plugin [83], and absolute PCC values used were those reported in literature [66].

Scratch wound healing assay was performed as reported [79].

### 3.3. Statistical Analyses

The statistical analyses of the data obtained from the various experiments (at least 3 independent tests) were performed by two-way ANOVA followed by Duncan’s post hoc test. The ANOVA test was performed considering data with a *p*-value less than 0.05 as significant. Data normality was tested by the Shapiro–Wilk test [84].

## 4. Conclusions

We have reported the synthesis of a nanosystem in which a new BODIPY, once activated, has been bound to novel amphiphilic NPs, thus obtaining water-soluble PSs. Considering that the solubilization of the free BODIPY in biological media is achievable only in the presence of organic solvents, this new nanosystem could be used for in vivo applications. Furthermore, the NP-bounded derivatives showed interesting chemical–physical features that could make them good PSs, such as significantly higher photostability than the free BODIPY, with no intrinsic toxicity, and effective anti-tumor photodynamic activity on two solid tumor cell lines at nanomolar concentrations. However, their potency was significantly lower compared to that of the free BODIPY, probably attributable to its lower cellular penetration. In addition, although the NP-bounded and free BODIPY shared the same lysosomal localization, the preferential mechanisms of cell death following PDT, among the ones investigated in the present work, are different. Finally, another interesting finding is that both BODIPY formulations significantly impaired cell migration of the SKOV3 cell line following PDT.

Despite the better photodynamic effects of the free form of BODIPY observed in these 2D models, the use of the new nanosystem in 3D models and in vivo in animal models should take advantage of its better solubility in aqueous solvents, meaning better in vivo bioavailability, and of the EPR effect, probably showing increased potency.

Taken together, our results indicate an interesting potential of these NP-bounded BODIPYs, which are worthy of further in-depth studies for their possible in vivo application.

## Data Availability

Data are contained within the article and Supplementary Materials.

## Supplementary Materials

The following supporting information can be downloaded at: https://www.mdpi.com/article/10.3390/ijms25063187/s1.

## References

[1] Zhao X., Liu J., Fan J., Chao H., Peng X. (2021). Recent progress in photosensitizers for overcoming the challenges of photodynamic therapy: From molecular design to application. Chem. Soc. Rev..

[2] Kwiatkowski S., Knap B., Przystupski D., Saczko J., Kedzierska E., Knap-Czop K., Kotlinska J., Michel O., Kotowski K., Kulbacka J. (2018). Photodynamic therapy—Mechanisms, photosensitizers and combinations. Biomed. Pharmacother..

[3] Zhang Q., Li L. (2018). Photodynamic combinational therapy in cancer treatment. J. BUON.

[4] Karolin J., Johansson L.B.A., Strandberg L., Ny T. (2002). Fluorescence and Absorption Spectroscopic Properties of Dipyrrometheneboron Difluoride (BODIPY) Derivatives in Liquids, Lipid Membranes, and Proteins. J. Am. Chem. Soc..

[5] Wittmershaus B.P., Skibicki J.J., McLafferty J.B., Zhang Y.Z., Swan S. (2001). Spectral properties of single BODIPY dyes in polystyrene microspheres and in solutions. J. Fluoresc..

[6] Banfi S., Nasini G., Zaza S., Caruso E. (2013). Synthesis and photo-physical properties of a series of BODIPY dyes. Tetrahedron.

[7] Boens N., Leen V., Dehaen W. (2012). Fluorescent indicators based on BODIPY. Chem. Soc. Rev..

[8] Golovkova T.A., Kozlov D.V., Neckers D.C. (2005). Synthesis and Properties of Novel Fluorescent Switches. J. Org. Chem..

[9] Miele Y., Mingotaud A.F., Caruso E., Malacarne M.C., Izzo L., Lonetti B., Rossi F. (2021). Hybrid giant lipid vesicles incorporating a PMMA-based copolymer. Biochim. Biophys. Acta Gen. Subj..

[10] Zhao N., Williams T.M., Zhou Z., Fronczek F.R., Sibrian-Vazquez M., Jois S.D., Vicente M.G.H. (2017). Synthesis of BODIPY-Peptide Conjugates for Fluorescence Labeling of EGFR Overexpressing Cells. Bioconjugate Chem..

[11] Caruso E., Orlandi V.T., Malacarne M.C., Martegani E., Scanferla C., Pappalardo D., Vigliotta G., Izzo L. (2021). Bodipy-Loaded Micelles Based on Polylactide as Surface Coating for Photodynamic Control of Staphylococcus aureus. Coatings.

[12] Malacarne M.C., Banfi S., Rugiero M., Caruso E. (2021). Drug delivery systems for the photodynamic application of two photosensitizers belonging to the porphyrin family. Photochem. Photobiol. Sci..

[13] Berg K., Golab J., Korbelik M., Russell D. (2011). Drug delivery technologies and immunological aspects of photodynamic therapy. Photochem. Photobiol. Sci..

[14] Wöhrle D., Hirth A., Bogdahn-Rai T., Schnurpfeil G., Shopova M. (1998). Photodynamic therapy of cancer: Second and third generations of photosensitizers. Russ. Chem. Bull..

[15] Josefsen L.B., Boyle R.W. (2012). Unique diagnostic and therapeutic roles of porphyrins and phthalocyanines in photodynamic therapy, imaging and theranostics. Theranostics.

[16] Lim C.K., Heo J., Shin S., Jeong K., Seo Y.H., Jang W.D., Park C.R., Park S.Y., Kim S., Kwon I.C. (2013). Nanophotosensitizers toward advanced photodynamic therapy of Cancer. Cancer Lett..

[17] Izci M., Maksoudian C., Manshian B.B., Soenen S.J. (2021). The Use of Alternative Strategies for Enhanced Nanoparticle Delivery to Solid Tumors. Chem. Rev..

[18] Prabhakar U., Maeda H., Jain R.K., Sevick-Muraca E.M., Zamboni W., Farokhzad O.C., Barry S.T., Gabizon A., Grodzinski P., Blakey D.C. (2013). Challenges and Key Considerations of the Enhanced Permeability and Retention Effect for Nanomedicine Drug Delivery in Oncology. Cancer Res..

[19] Wu J. (2021). The Enhanced Permeability and Retention (EPR) Effect: The Significance of the Concept and Methods to Enhance Its Application. J. Pers. Med..

[20] Dost Z., Atilgan S., Akkaya E.U. (2006). Distyryl-boradiazaindacenes: Facile synthesis of novel near IR emitting fluorophores. Tetrahedron.

[21] Liu J.Y., Yeung H.S., Xu W., Li X., Ng D.K. (2008). Highly efficient energy transfer in subphthalocyanine-BODIPY conjugates. Org. Lett..

[22] Malacarne M.C., Gariboldi M.B., Caruso E. (2022). BODIPYs in PDT: A Journey through the Most Interesting Molecules Produced in the Last 10 Years. Int. J. Mol. Sci..

[23] Ferguson C.T.J., Huber N., Landfester K., Zhang K.A.I. (2019). Dual-Responsive Photocatalytic Polymer Nanogels. Angew. Chem. Int. Ed. Engl..

[24] Wan J., Fan B., Thang S.H. (2022). RAFT-mediated polymerization-induced self-assembly (RAFT-PISA): Current status and future directions. Chem. Sci..

[25] Li R., Heuer J., Kuckhoff T., Landfester K., Ferguson C.T.J. (2023). pH-Triggered Recovery of Organic Polymer Photocatalytic Particles for the Production of High Value Compounds and Enhanced Recyclability. Angew. Chem. Int. Ed. Engl..

[26] Aydin D.Y., Gürü M., Akkurt F. (2021). Investigation of Synthesis Parameters of Antimony Fluoroborate and Its Usability as a Flame Retardant for Cellulosic Fabrics. Cell Chem. Technol..

[27] Bonnett R., Martínez G. (2001). Photobleaching of sensitisers used in photodynamic therapy. Tetrahedron.

[28] Georgakoudi I., Foster T.H. (1998). Singlet oxygen- versus nonsinglet oxygen-mediated mechanisms of sensitizer photobleaching and their effects on photodynamic dosimetry. Photochem. Photobiol..

[29] Kochevar I.E., Redmond R.W. (2000). Photosensitized production of singlet oxygen. Methods in Enzymology.

[30] Rodríguez-Bonilla P., Gandía-Herrero F., Matencio A., García-Carmona F., López-Nicolás J.M. (2017). Comparative Study of the Antioxidant Capacity of Four Stilbenes Using ORAC, ABTS+, and FRAP Techniques. Food Anal. Methods.

[31] Gullcin I., Dastan A. (2007). Synthesis of dimeric phenol derivatives and determination of in vitro antioxidant and radical scavenging activities. J. Enzym. Inhib. Med. Chem..

[32] Enko B., Borisov S.M., Regensburger J., Baumler W., Gescheidt G., Klimant I. (2013). Singlet oxygen-induced photodegradation of the polymers and dyes in optical sensing materials and the effect of stabilizers on these processes. J. Phys. Chem. A.

[33] Fang J., Islam W., Maeda H. (2020). Exploiting the dynamics of the EPR effect and strategies to improve the therapeutic effects of nanomedicines by using EPR effect enhancers. Adv. Drug Deliv. Rev..

[34] Rai A., Ferreira L. (2021). Biomedical applications of the peptide decorated gold nanoparticles. Crit. Rev. Biotechnol..

[35] Mfouo-Tynga I.S., Dias L.D., Inada N.M., Kurachi C. (2021). Features of third generation photosensitizers used in anticancer photodynamic therapy: Review. Photodiagnosis Photodyn. Ther..

[36] Niculescu A.G., Grumezescu A.M. (2021). Photodynamic Therapy-An Up-to-Date Review. Appl. Sci..

[37] Pandey V., Raza M.K., Joshi P., Gupta I. (2020). Synthesis of Water-Soluble Thioglycosylated trans-A(2)B(2) Type Porphyrins: Cellular Uptake Studies and Photodynamic Efficiency. J. Org. Chem..

[38] Ng S.Y., Kamkaew A., Fu N., Kue C.S., Chung L.Y., Kiew L.V., Wittayakun J., Burgess K., Lee H.B. (2020). Active targeted ligand-aza-BODIPY conjugate for near-infrared photodynamic therapy in melanoma. Int. J. Pharm..

[39] Lamch L., Bazylinska U., Kulbacka J., Pietkiewicz J., Biezunska-Kusiak K., Wilk K.A. (2014). Polymeric micelles for enhanced Photofrin II (R) delivery, cytotoxicity and pro-apoptotic activity in human breast and ovarian cancer cells. Photodiagnosis Photodyn. Ther..

[40] Al Musaimi O., Al Shaer D., de la Torre B.G., Albericio F. (2018). 2017 FDA Peptide Harvest. Pharmaceuticals.

[41] Kim J., Park W., Kim D., Lee E.S., Lee D.H., Jeong S., Park J.M., Na K. (2019). Tumor-Specific Aptamer-Conjugated Polymeric Photosensitizer for Effective Endo-Laparoscopic Photodynamic Therapy. Adv. Funct. Mater..

[42] Nam K.C., Han Y.S., Lee J.M., Kim S.C., Cho G., Park B.J. (2020). Photo-Functionalized Magnetic Nanoparticles as a Nanocarrier of Photodynamic Anticancer Agent for Biomedical Theragnostics. Cancers.

[43] Ofir R., Seidman R., Rabinski T., Krup M., Yavelsky V., Weinstein Y., Wolfson M. (2002). Taxol-induced apoptosis in human SKOV3 ovarian and MCF7 breast carcinoma cells is caspase-3 and caspase-9 independent. Cell Death Differ..

[44] Ballestri M., Caruso E., Guerrini A., Ferroni C., Banfi S., Gariboldi M., Monti E., Sotgiu G., Varchi G. (2018). Core-shell poly-methyl methacrylate nanoparticles covalently functionalized with a non-symmetric porphyrin for anticancer photodynamic therapy. J. Photochem. Photobiol. B.

[45] Heinemann F., Karges J., Gasser G. (2017). Critical Overview of the Use of Ru(II) Polypyridyl Complexes as Photosensitizers in One-Photon and Two-Photon Photodynamic Therapy. Acc. Chem. Res..

[46] Brillo V., Chieregato L., Leanza L., Muccioli S., Costa R. (2021). Mitochondrial Dynamics, ROS, and Cell Signaling: A Blended Overview. Life.

[47] Gomes A., Fernandes E., Lima J.L. (2005). Fluorescence probes used for detection of reactive oxygen species. J. Biochem. Biophys. Methods.

[48] Aranda A., Sequedo L., Tolosa L., Quintas G., Burello E., Castell J.V., Gombau L. (2013). Dichloro-dihydro-fluorescein diacetate (DCFH-DA) assay: A quantitative method for oxidative stress assessment of nanoparticle-treated cells. Toxicol. Vitr..

[49] Kessler A., Hedberg J., McCarrick S., Karlsson H.L., Blomberg E., Odnevall I. (2021). Adsorption of Horseradish Peroxidase on Metallic Nanoparticles: Effects on Reactive Oxygen Species Detection Using 2’,7’-Dichlorofluorescin Diacetate. Chem. Res. Toxicol..

[50] Kim S., Lee T.J., Leem J., Choi K.S., Park J.W., Kwon T.K. (2008). Sanguinarine-induced apoptosis: Generation of ROS, down-regulation of Bcl-2, c-FLIP, and synergy with TRAIL. J. Cell Biochem..

[51] Wang L., Azad N., Kongkaneramit L., Chen F., Lu Y., Jiang B.H., Rojanasakul Y. (2008). The Fas death signaling pathway connecting reactive oxygen species generation and FLICE inhibitory protein down-regulation. J. Immunol..

[52] Liu Y., Borchert G.L., Surazynski A., Hu C.A., Phang J.M. (2006). Proline oxidase activates both intrinsic and extrinsic pathways for apoptosis: The role of ROS/superoxides, NFAT and MEK/ERK signaling. Oncogene.

[53] Chen P., Luo X., Nie P., Wu B., Xu W., Shi X., Chang H., Li B., Yu X., Zou Z. (2017). CQ synergistically sensitizes human colorectal cancer cells to SN-38/CPT-11 through lysosomal and mitochondrial apoptotic pathway via p53-ROS cross-talk. Free Radic. Biol. Med..

[54] Zuo Y., Xiang B., Yang J., Sun X., Wang Y., Cang H., Yi J. (2009). Oxidative modification of caspase-9 facilitates its activation via disulfide-mediated interaction with Apaf-1. Cell Res..

[55] Luanpitpong S., Chanvorachote P., Stehlik C., Tse W., Callery P.S., Wang L., Rojanasakul Y. (2013). Regulation of apoptosis by Bcl-2 cysteine oxidation in human lung epithelial cells. Mol. Biol. Cell.

[56] Zhang Y., Su S.S., Zhao S., Yang Z., Zhong C.-Q., Chen X., Cai Q., Yang Z.-H., Huang D., Wu R. (2017). RIP1 autophosphorylation is promoted by mitochondrial ROS and is essential for RIP3 recruitment into necrosome. Nat. Commun..

[57] Sun W., Wu X., Gao H., Yu J., Zhao W., Lu J.J., Wang J., Du G., Chen X. (2017). Cytosolic calcium mediates RIP1/RIP3 complex-dependent necroptosis through JNK activation and mitochondrial ROS production in human colon cancer cells. Free Radic. Biol. Med..

[58] Zhou Z., Lu B., Wang C., Wang Z., Luo T., Piao M., Meng F., Chi G., Luo Y., Ge P. (2017). RIP1 and RIP3 contribute to shikonin-induced DNA double-strand breaks in glioma cells via increase of intracellular reactive oxygen species. Cancer Lett..

[59] Zhao Y., Qu T., Wang P., Li X., Qiang J., Xia Z., Duan H., Huang J., Zhu L. (2016). Unravelling the relationship between macroautophagy and mitochondrial ROS in cancer therapy. Apoptosis.

[60] Scherz-Shouval R., Shvets E., Fass E., Shorer H., Gil L., Elazar Z. (2007). Reactive oxygen species are essential for autophagy and specifically regulate the activity of Atg4. Embo J..

[61] Song C., Mitter S.K., Qi X., Beli E., Rao H.V., Ding J., Ip C.S., Gu H., Akin D., Dunn W.A. (2017). Oxidative stress-mediated NFkappaB phosphorylation upregulates p62/SQSTM1 and promotes retinal pigmented epithelial cell survival through increased autophagy. PLoS ONE.

[62] Kessel D. (2019). Apoptosis, Paraptosis and Autophagy: Death and Survival Pathways Associated with Photodynamic Therapy. Photochem. Photobiol..

[63] Kessel D., Reiners J.J. (2020). Photodynamic therapy: Autophagy and mitophagy, apoptosis and paraptosis. Autophagy.

[64] Mishchenko T., Balalaeva I., Gorokhova A., Vedunova M., Krysko D.V. (2022). Which cell death modality wins the contest for photodynamic therapy of cancer?. Cell Death Dis..

[65] Ouedraogo G.D., Redmond R.W. (2003). Secondary reactive oxygen species extend the range of photosensitization effects in cells: DNA damage produced via initial membrane photosensitization. Photochem. Photobiol..

[66] Adler J., Parmryd I. (2010). Quantifying colocalization by correlation: The Pearson correlation coefficient is superior to the Mander’s overlap coefficient. Cytom. A.

[67] Moor A.C. (2000). Signaling pathways in cell death and survival after photodynamic therapy. J. Photochem. Photobiol. B.

[68] Tsubone T.M., Martins W.K., Pavani C., Junqueira H.C., Itri R., Baptista M.S. (2017). Enhanced efficiency of cell death by lysosome-specific photodamage. Sci. Rep..

[69] Lee S.J., Bae J.H., Lee A.W., Tong S.Y., Park Y.G., Park J.S. (2009). Clinical characteristics of metastatic tumors to the ovaries. J. Korean Med. Sci..

[70] Coffman L.G., Burgos-Ojeda D., Wu R., Cho K., Bai S., Buckanovich R.J. (2016). New models of hematogenous ovarian cancer metastasis demonstrate preferential spread to the ovary and a requirement for the ovary for abdominal dissemination. Transl. Res..

[71] Ma R., Ye X., Cheng H., Cui H., Chang X. (2021). Tumor-derived exosomal circRNA051239 promotes proliferation and migration of epithelial ovarian cancer. Am. J. Transl. Res..

[72] Yousefi M., Dehghani S., Nosrati R., Ghanei M., Salmaninejad A., Rajaie S., Hasanzadeh M., Pasdar A. (2020). Current insights into the metastasis of epithelial ovarian cancer—Hopes and hurdles. Cell Oncol..

[73] Hergueta-Redondo M., Peinado H. (2020). The influence of secreted factors and extracellular vesicles in ovarian cancer metastasis. EJC Suppl..

[74] Wang X., Hu J., Wang P., Zhang S., Liu Y., Xiong W., Liu Q. (2015). Analysis of the in vivo and in vitro effects of photodynamic therapy on breast cancer by using a sensitizer, sinoporphyrin sodium. Theranostics.

[75] Gheewala T., Skwor T., Munirathinam G. (2018). Photodynamic therapy using pheophorbide and 670nm LEDs exhibits anti-cancer effects in-vitro in androgen dependent prostate cancer. Photodiagnosis Photodyn. Ther..

[76] Du S.W., Zhang L.K., Han K., Chen S., Hu Z., Chen W., Hu K., Yin L., Wu B., Guan Y.Q. (2018). Combined Phycocyanin and Hematoporphyrin Monomethyl Ether for Breast Cancer Treatment via Photosensitizers Modified Fe(3)O(4) Nanoparticles Inhibiting the Proliferation and Migration of MCF-7 Cells. Biomacromolecules.

[77] Jiang J., Wang K., Chen Y., Chen H., Nice E.C., Huang C. (2017). Redox regulation in tumor cell epithelial-mesenchymal transition: Molecular basis and therapeutic strategy. Signal Transduct. Target. Ther..

[78] https://www.cellbiolabs.com/sites/default/files/CBA-120-wound-healing-assay_0.pdf.

[79] Caruso E., Cerbara M., Malacarne M.C., Marras E., Monti E., Gariboldi M.B. (2019). Synthesis and photodynamic activity of novel non-symmetrical diaryl porphyrins against cancer cell lines. J. Photochem. Photobiol. B Biol..

[80] Huang L., Lin H., Chen Q., Yu L., Bai D. (2019). MPPa-PDT suppresses breast tumor migration/invasion by inhibiting Akt-NF-kappaB-dependent MMP-9 expression via ROS. BMC Cancer.

[81] Yoshida A., Yoshino F., Makita T., Maehata Y., Higashi K., Miyamoto C., Wada-Takahashi S., Takahashi S.S., Takahashi O., Lee M.C. (2013). Reactive oxygen species production in mitochondria of human gingival fibroblast induced by blue light irradiation. J. Photochem. Photobiol. B Biol..

[82] Zagami R., Sortino G., Caruso E., Malacarne M.C., Banfi S., Patane S., Monsu Scolaro L., Mazzaglia A. (2018). Tailored-BODIPY/Amphiphilic Cyclodextrin Nanoassemblies with PDT Effectiveness. Langmuir.

[83] Schindelin J., Arganda-Carreras I., Frise E., Kaynig V., Longair M., Pietzsch T., Preibisch S., Rueden C., Saalfeld S., Schmid B. (2012). Fiji: An open-source platform for biological-image analysis. Nat. Methods.

[84] Tabachnick B.G., Fidell L.S. (2019). Using Multivariate Statistics.

