# Supplementary material for: Evaluation of Nanoparticles Covalently Bound with BODIPY for Their Photodynamic Therapy Applicability
Source: Int J Mol Sci. 2024 Mar 10;25(6):3187. doi: 10.3390/ijms25063187 (PMC10969874; doi:10.3390/ijms25063187)
Supplement: Supplementary file 1 [file ijms-25-03187-s001.zip › ijms-2680559-supplementary.pdf]

Supporting information for:

Evaluation of Nanoparticles Covalently Bound with BODIPY for Their Photodynamic Therapy Applicability

Miryam Chiara Malacarne [1], Enrico Caruso [1], Marzia Bruna Gariboldi [1], Emanuela Marras [1], Gianluca Della Bitta [1], Orlando Santoro [1,*], Alan Simm [2], Rong Li [3] and Calum T. J. Ferguson [3]

[1] Department of Biotechnology and Life Sciences (DBSV), University of Insubria, Via J.H. Dunant 3, 21100 Varese, Italy; mc.malacarne1@uninsubria.it (M.C.M.); enrico.caruso@uninsubria.it (E.C.); marzia.gariboldi@uninsubria.it (M.B.G.); emanuela.marras@uninsubria.it (E.M.); gianluca.dellabitta@libero.it (G.D.B.)

[2] Faculty of Sciences, Byrom Street Campus, Liverpool John Moores University, Liverpool L3 3AF, UK; a.m.simm@ljmu.ac.uk

[3] Max Planck Institute for Polymer Research, Ackermannweg 10, 55128 Mainz, Germany; lirong@mpip-mainz.mpg.de (R.L.); ferguson@mpip-mainz.mpg.de (C.T.J.F.)

* Correspondence: orlando.santoro@uninsubria.it; Tel.: +39-0332421310

Contents

Figure S1. GPC spectra of LIR-10-14B (Mn = 34.6 kDa, red) and LIR-10-15 (Mn = 42.3 kDa, dark grey). 3

Figure S2. ^{1}H NMR spectrum of LIR-10-14B in DMSO-d6 (300 MHz, at 298 K). 4

Figure S3. ^{1}H NMR spectrum of LIR-10-15 in DMSO-d6 (300 MHz, at 298 K). 5

Figure S4. FTIR spectra of LIR-10-15 (dark grey) and LIR-10-14B (red). The FTIR spectra were performed with KBr pellets. The C-H/C-C backbone vibration peaks were observed in the range of 3130-2775 cm^{-1}. The C=O vibration peaked at 1727 cm^{-1} assigned to acrylate bonds. The Ar-O stretching was observed at 1262 cm^{-1} belonging to alkyl aryl ether bonds from BODIPY section. The C-O stretching ascribed to acrylate linkages appeared at 1174 cm^{-1}. Besides, the B-F vibration was observed at 1055 cm^{-1}. 6

Figure S5. Residual stability of 10 μM solution of PSs in PBS 1X after 500 W tungsten halogen lamp irradiation for 2h and subjected to spectrophotometric analysis (**BOD**: full bar; **15** (**BOD**): striped bar). 7

Figure S6. Survival rate of cells in the absence of irradiation and after 24h of treatment with a PS concentration corresponding to ten times (1000 nM for **BOD-I$_2$**, 20 μM for **14B**, and 10 μM for **15**) the maximum concentration used during the PDT experiments (**MCF7**: dark blue; **SKOV3**: pale blue). Mean ± SD of 3 independent experiments. 8

Figure S7. Dose-response curves in MCF7 (**A**) and SKOV3 (**A**) cells (**BOD-I$_2$**: pink, **14B**: dark blue, **15**: bright blue**)**. Cells were grown for 48h prior to 24h treatment with PSs, 2h irradiation under visible light of a 500 W halogen lamp and additional 24h incubation in drug-free medium. The effect of PSs on cell viability was determined using the MTT assay. Mean ± SD of 5 independent experiments. 9

Figure S8. Survival rate of MRC-5 cells after 24h treatment with PSs (1000 to 1 nM), 2h irradiation under visible light of a 500 W halogen lamp, 24h incubation in drug-free medium and MTT assay (**BOD-I$_2$**: dark green; **14B**: green; **15**: pale green). Mean ± SD of 3 independent experiments. 10

Figure S9. Fluorescent microscopy images of ROS generation in cells (magnification 4X). Cells are seeded in a black 96-well plate, treated with PS at the respective IC_{50} for 24h, and irradiated for 2h in PS-free PBS. After irradiation, cells are washed with PBS, and 10 μM of DCFH-DA was added to each well. Cells are then incubated in the dark at 37 °C for 30 min. The data are analysed by fluorescence intensity and normalized to control group. Fluorescence microscopy was used to detect the production of ROS (Ex: 488 nm; Em: 520 nm). For control samples, treatment with PS is omitted. The ROS production rate, expressed as arbitrary fluorescence units, is measured with ImageJ software. 11

Figure S10. Migratory activity of SKOV3 cells following treatment with **BOD-I$_2$**, **14B, 15.** Cells are seeded in a 6-well plate and allowed to grow for 48h to reach confluence and treated with the IC_{25} concentrations of the compounds. After 24h, in each well a scratch is performed with a pipette tip. Cells are placed in drug-free medium for 24h. Pictures of the scratch wound were taken immediately following the wound making and after 24h, through a camera connected to an Olympus IX81 microscope (Magnification 4X). 12

Figure S1. GPC spectra of LIR-10-14B (Mn = 34.6 kDa, red) and LIR-10-15 (Mn = 42.3 kDa, dark grey).

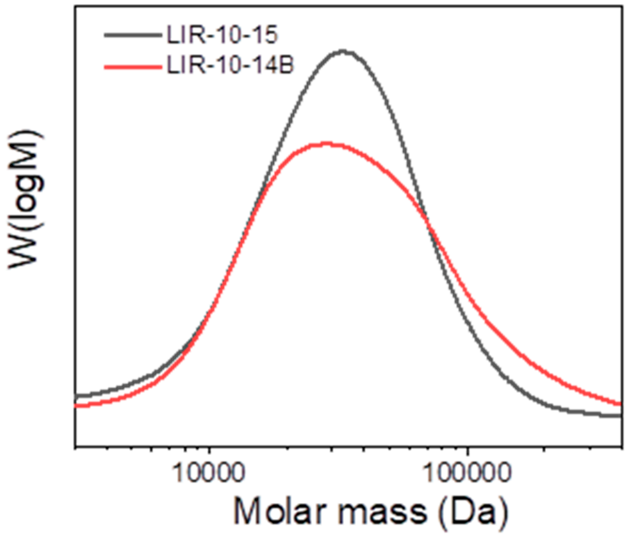

Figure S2. ^{1}H NMR spectrum of LIR-10-14B in DMSO-d6 (300 MHz, at 298 K).

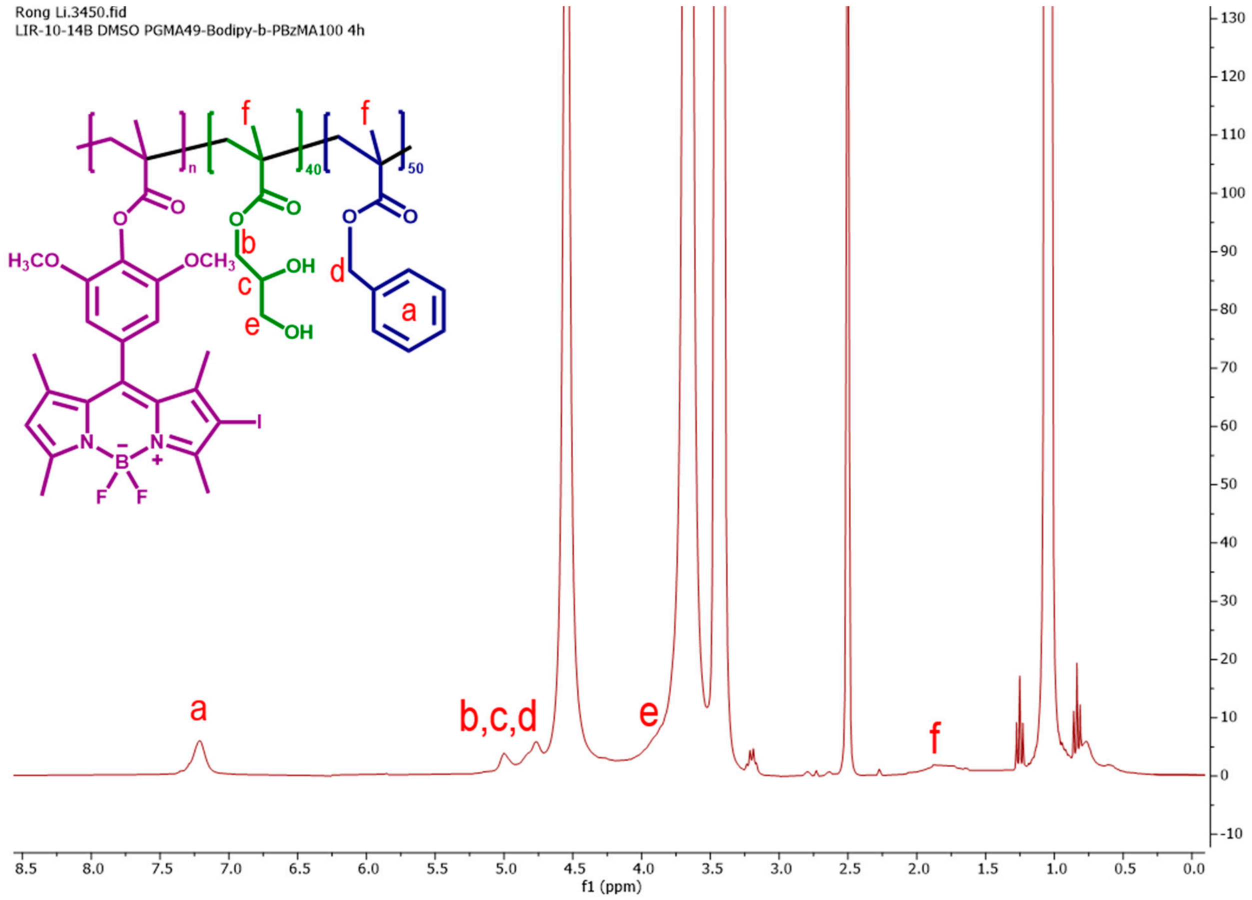

Figure S3. ^{1}H NMR spectrum of LIR-10-15 in DMSO-d6 (300 MHz, at 298 K).

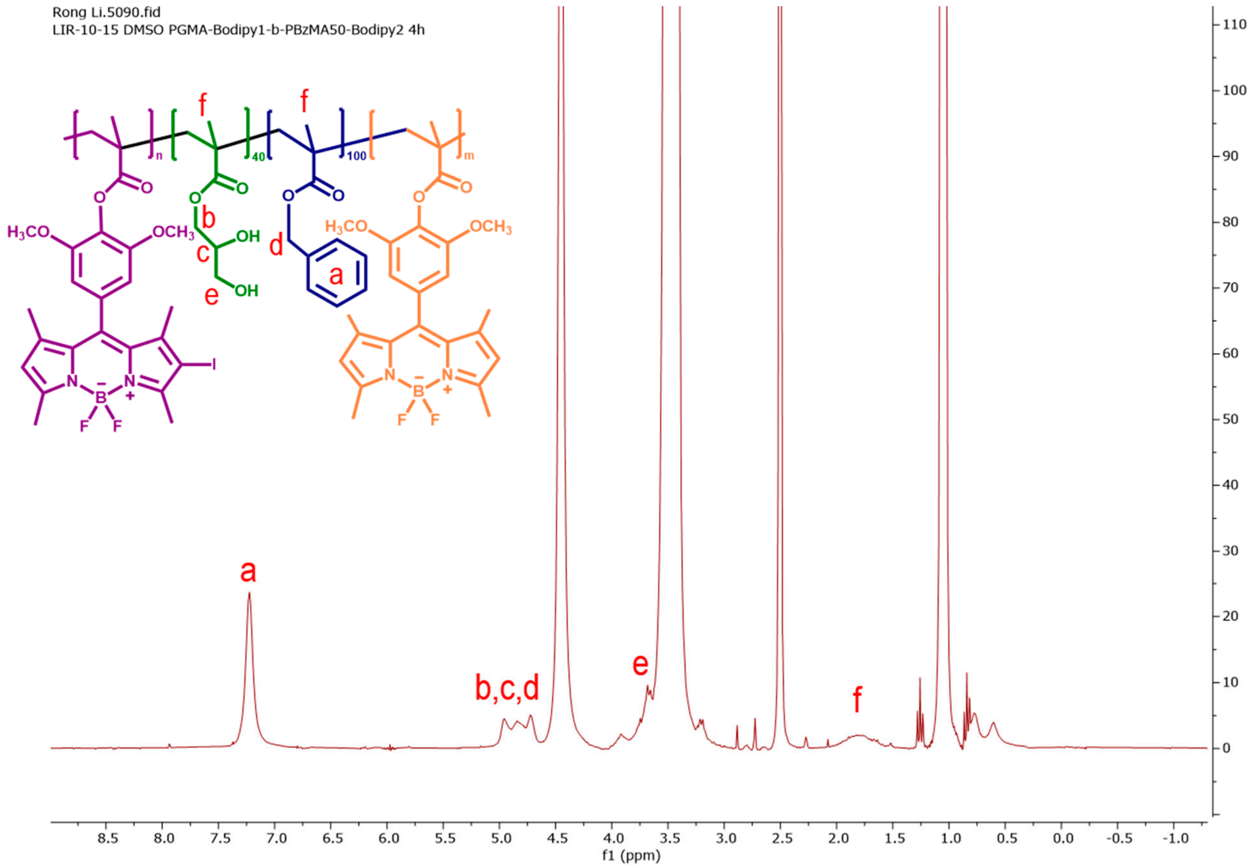

Figure S4. FTIR spectra of LIR-10-15 (dark grey) and LIR-10-14B (red). The FTIR spectra were performed with KBr pellets. The C-H/C-C backbone vibration peaks were observed in the range of 3130-2775 cm^{-1}. The C=O vibration peaked at 1727 cm^{-1} assigned to acrylate bonds. The Ar-O stretching was observed at 1262 cm^{-1} belonging to alkyl aryl ether bonds from BODIPY section. The C-O stretching ascribed to acrylate linkages appeared at 1174 cm^{-1}. Besides, the B-F vibration was observed at 1055 cm^{-1}.

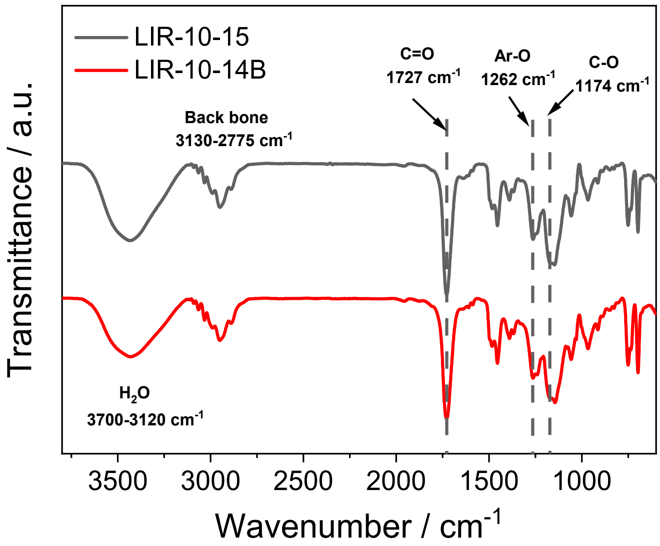

Figure S5. Residual stability of 10 μM solution of PSs in PBS 1X after 500 W tungsten halogen lamp irradiation for 2h and subjected to spectrophotometric analysis (**BOD**: full bar; **15** (**BOD**): striped bar).

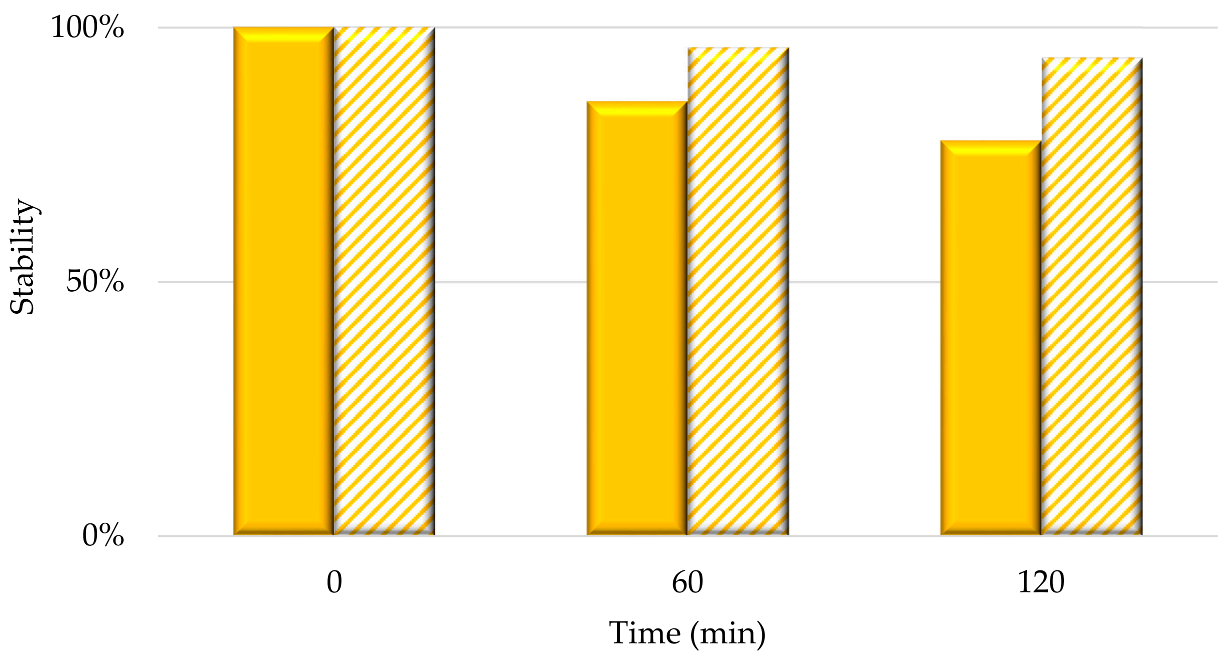

Figure S6. Survival rate of cells in the absence of irradiation and after 24h of treatment with a PS concentration corresponding to ten times (1000 nM for **BOD-I₂**, 20 μM for **14B**, and 10 μM for **15**) the maximum concentration used during the PDT experiments (**MCF7**: dark blue; **SKOV3**: pale blue). Mean ± SD of 3 independent experiments.

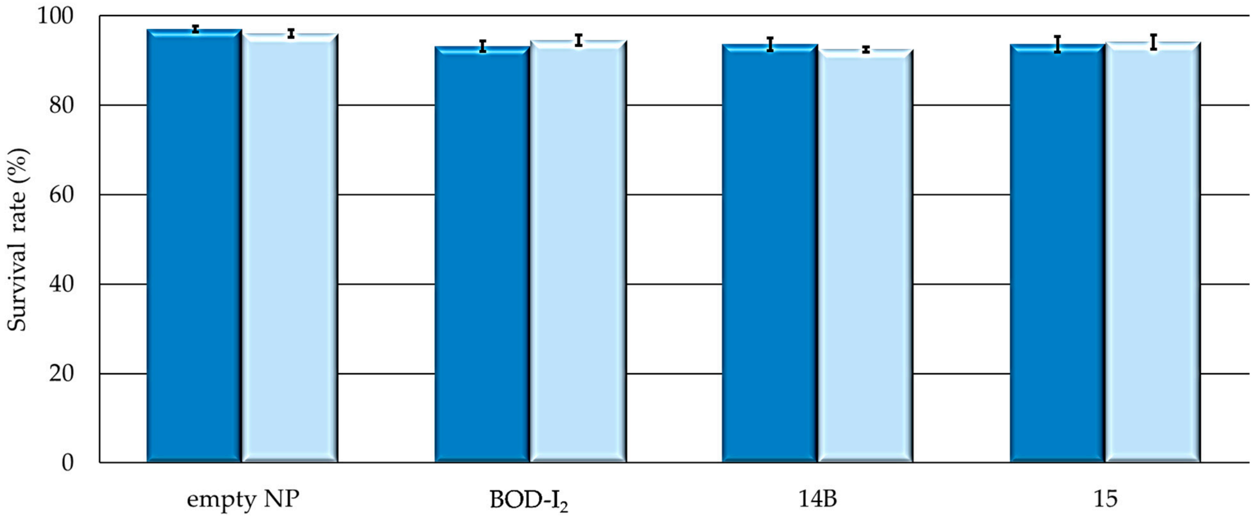

Figure S7. Dose-response curves in MCF7 (**A**) and SKOV3 (**B**) cells (**BOD-I**$_2$: pink, **14B**: dark blue, **15**: bright blue**)**. Cells were grown for 48h prior to 24h treatment with PSs, 2h irradiation under visible light of a 500 W halogen lamp and additional 24h incubation in drug-free medium. The effect of PSs on cell viability was determined using the MTT assay. Mean ± SD of 5 independent experiments.

A

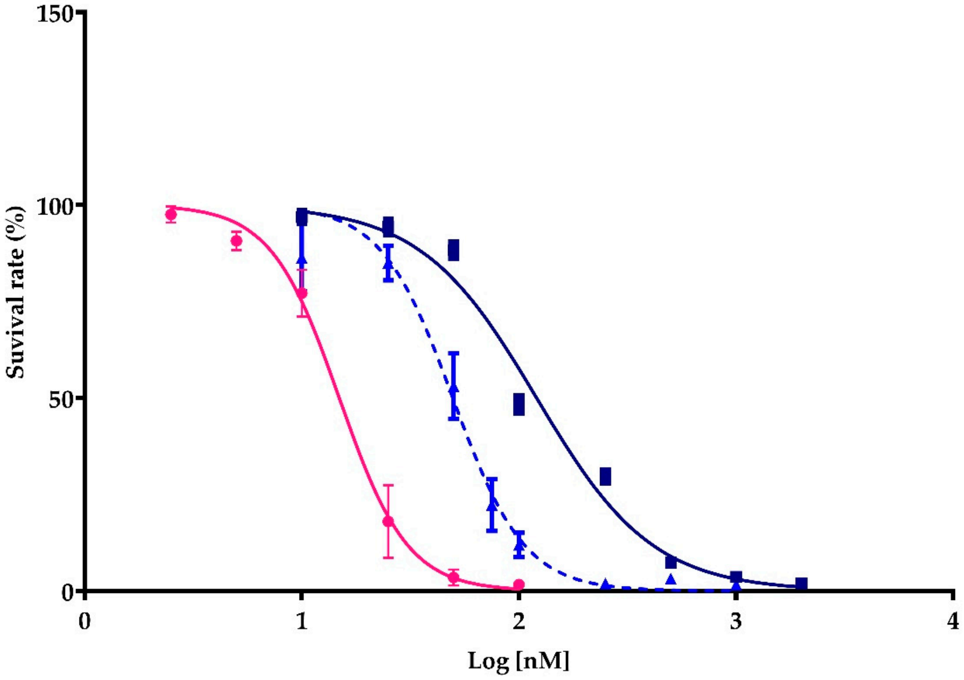

B

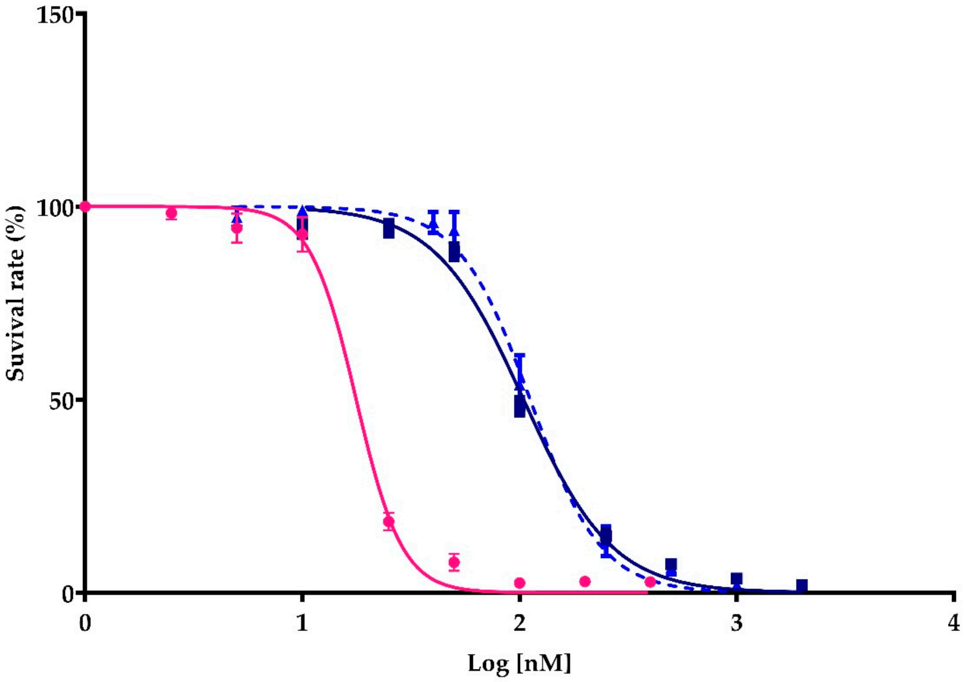

Figure S8. Survival rate of MRC-5 cells after 24h treatment with PSs (1000 to 1 nM), 2h irradiation under visible light of a 500 W halogen lamp, 24h incubation in drug-free medium and MTT assay (**BOD-I$_2$**: dark green; **14B**: green; **15**: pale green). Mean ± SD of 3 independent experiments.

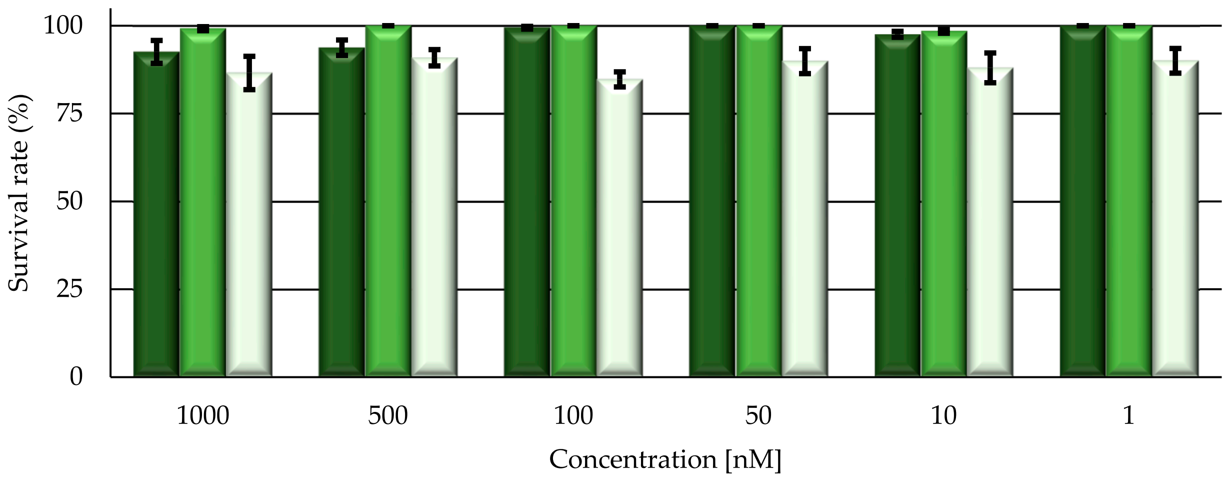

Figure S9. Fluorescent microscopy images of ROS generation in cells (magnification 4X). Cells are seeded in a black 96-well plate, treated with PS at the respective IC_{50} for 24h, and irradiated for 2h in PS-free PBS. After irradiation, cells are washed with PBS, and 10 μM of DCFH-DA was added to each well. Cells are then incubated in the dark at 37 °C for 30 min. The data are analysed by fluorescence intensity and normalized to control group. Fluorescence microscopy was used to detect the production of ROS (Ex: 488 nm; Em: 520 nm). For control samples, treatment with PS is omitted. The ROS production rate, expressed as arbitrary fluorescence units, is measured with ImageJ software.

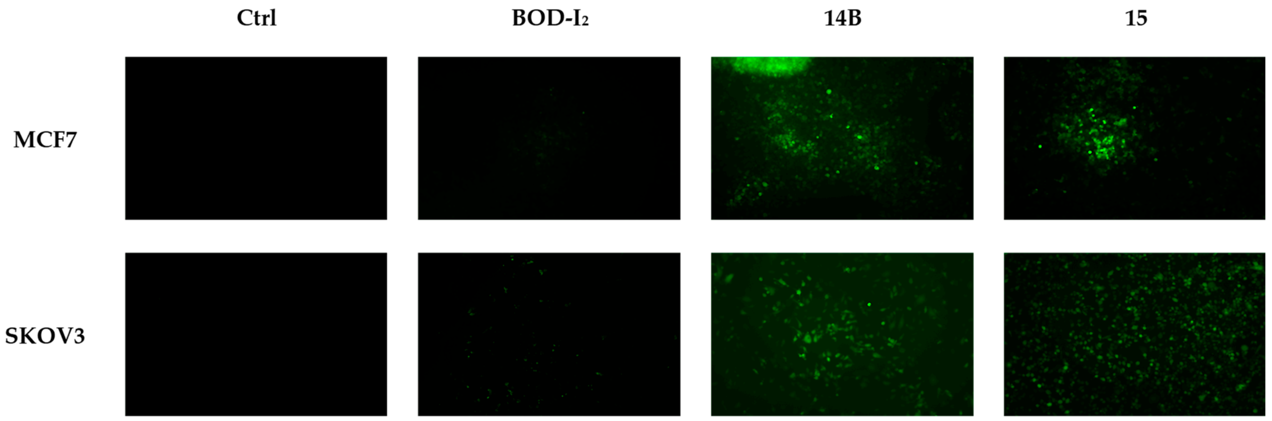

Figure S10. Migratory activity of SKOV3 cells following treatment with **BOD-I$_2$**, **14B, 15.** Cells are seeded in a 6-well plate and allowed to grow for 48h to reach confluence and treated with the IC_{25} concentrations of the compounds. After 24h, in each well a scratch is performed with a pipette tip. Cells are placed in drug-free medium for 24h. Pictures of the scratch wound were taken immediately following the wound making and after 24h, through a camera connected to an Olympus IX81 microscope (Magnification 4X).

	t_0	t_{24}	% of closure	Migration rate
**Ctrl**	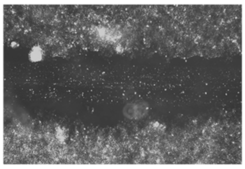	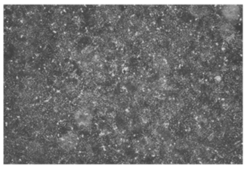	100.00	7.30
**BOD-I$_2$**	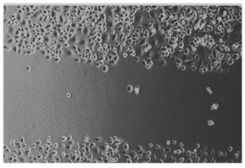	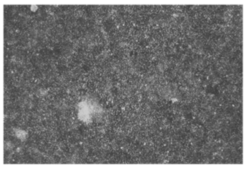	100.00	6.66
**14B**	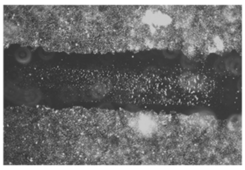	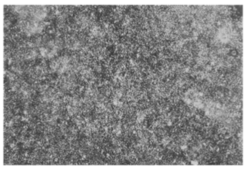	100.00	6.44
**15**	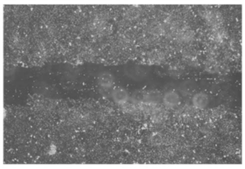	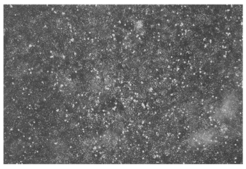	100.00	6.17
